# Nodular regenerative hyperplasia and liver transplantation: a systematic review

**DOI:** 10.3389/frtra.2023.1221765

**Published:** 2023-09-06

**Authors:** Ben E. Biesterveld, Paul M. Schroder, Mary E. Hitchcock, Alexandra Bolognese, Steven C. Kim, David P. Al-Adra

**Affiliations:** ^1^ Department of Transplant Surgery, University of Wisconsin, Madison, WI, United States; ^2^ Ebling Library, University of Wisconsin, Madison, WI, United States; ^3^ Division of Abdominal Organ Transplantation, Oregon Health & Science University School of Medicine, Portland, OR, United States; ^4^ Department of Surgery, Division of Transplantation, Emory University, Atlanta, GA, United States

**Keywords:** nodular regenerative hyperplasia, portal hypertension, ascites, encephalopathy, varices, biopsy

## Abstract

Nodular regenerative hyperplasia (NRH) is a primary disease of the liver that may cause noncirrhotic portal hypertension. Common causes include autoimmune, hematologic, immune deficiency, and myeloproliferative disorders. Given the limited data regarding the development of NRH in contemporary immunosuppressive protocols and the occurrence of NRH post-liver transplantation, we systematically reviewed NRH as it pertains to liver transplantation. We performed a comprehensive search for NRH and transplantation. Nineteen studies were identified with relevant data for NRH as an indication for a liver transplant. Thirteen studies were identified with relevant data pertaining to NRH development after liver transplant. Pooled analysis revealed 0.9% of liver transplant recipients had NRH. A total of 113 patients identified with NRH underwent liver transplantation. Most series report transplants done after the failure of endoscopic banding and TIPS management of portal hypertension. Reported 5-year graft and patient survival ranged from 73%–78% and 73%–90%. The pooled incidence of NRH after liver transplant for all indications was 2.9% and caused complications of portal hypertension. Complications related to portal hypertension secondary to NRH are a rare indication for a liver transplant. NRH can develop at any time after liver transplantation often without an identifiable cause, which may lead to portal hypertension requiring treatment or even re-transplantation.

## Introduction

Nodular regenerative hyperplasia (NRH) is a pathology of the liver characterized by the development of nodules throughout the liver parenchyma without the presence of background fibrosis. While this may be an indolent finding, in some cases, it can cause noncirrhotic portal hypertension and the associated complications of variceal bleeding, thrombocytopenia, ascites, encephalopathy, and hepatopulmonary syndrome (HPS). NRH is a rare clinical diagnosis with much of the literature limited to small cohorts and case series. However, autopsy studies have found a prevalence of 2.1%–2.6% in the general population (1, 2). Although the majority of cases of NRH have no known underlying etiology, it is associated with autoimmune, hematologic, immune deficiency, and myeloproliferative disorders (3–8).

NRH is also a recognized entity post-transplantation, with a multitude of case reports and case series of transplant recipients developing NRH after renal, heart, and bone marrow transplantation (9–12). Many of these series are historical as they were reported in an era with high utilization of azathioprine (AZA)-based immunosuppressive therapy. AZA exposure is associated with NRH development in transplant recipients as well as other populations such as those with inflammatory bowel disease (13). However, these limited reports of AZA-related NRH lack modern clinical relevance as contemporary immunosuppression regimens have shifted away from AZA.

The challenge of managing NRH is understudied and can cause significant clinical challenges. In fact, the stigmata of portal hypertension secondary to NRH may become severe enough that a liver transplant is indicated. In addition, NRH may develop after liver transplantation in the donor liver. Much earlier literature is limited to case reports and a systematic review of these case reports (3). Since then, several larger retrospective cohorts of patients transplanted for NRH have been published. Given the limited data regarding the development of NRH in contemporary immunosuppressive protocols and the occurrence of NRH post-liver transplantation, we systematically reviewed the literature on NRH as it pertains to liver transplantation. Specifically, this review focuses on NRH as the indication for liver transplant and the development of NRH in the transplanted liver.

## Methods

A systematic review of NRH and liver transplantation was conducted. Criteria for considering studies for this review included human case reports (1 case), case series (>1 case), randomized controlled trials, non-randomized controlled trials, case-control studies, and prospective and retrospective cohort series. The target population consisted of adult and pediatric male or female patients with a pathological diagnosis of NRH. All patients were diagnosed with NRH based on histology of a liver biopsy or final pathology of an explanted liver.

**Figure 1:**
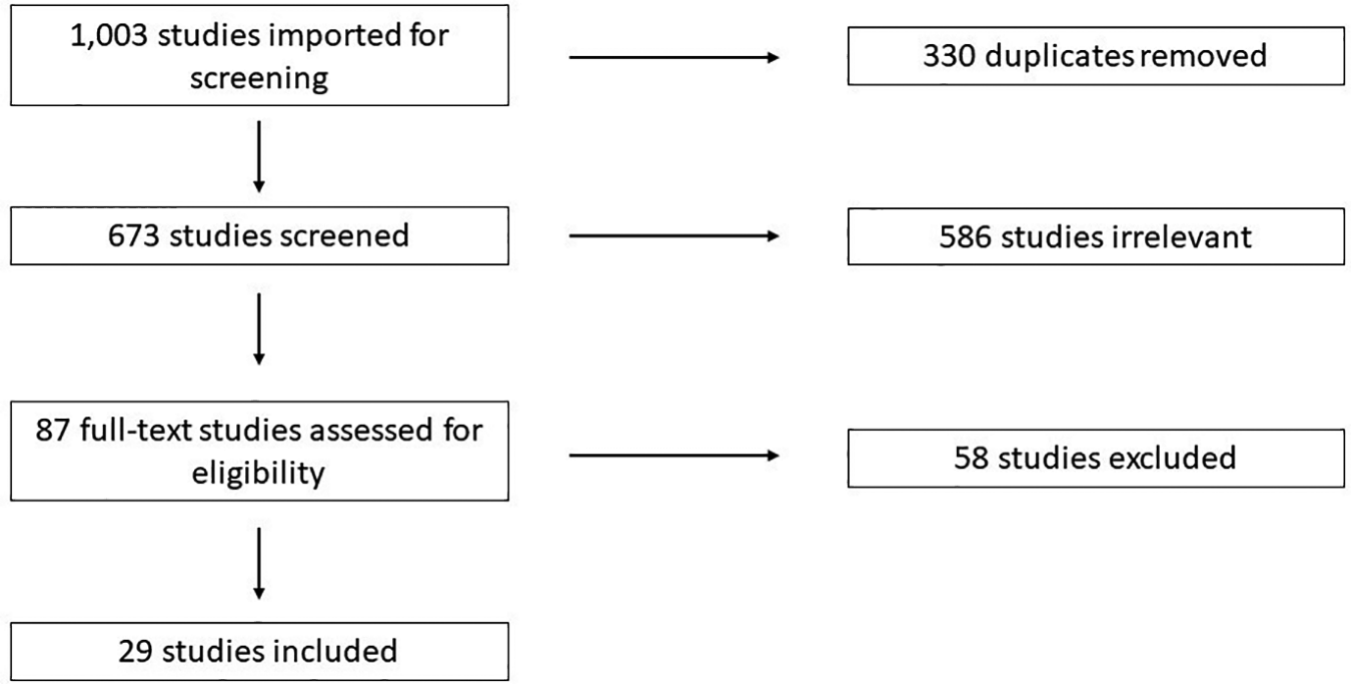
PRISMA flow diagram.

Search Terms: Studies were identified using a keyword search for relevant terms in PubMed, CINAHL, Scopus, Web of Science, and Cochrane. Search details are available in Supplementary File S1. References were imported into Covidence (https://www.covidence.org/) for screening. Studies were screened by three reviewers independently according to PRISMA guidelines (Figure 1).

## Results

### Search results

A total of 1,003 studies were imported for screening, and 330 duplicates were removed. Reviewers screened the title and abstract of 673 studies and 87 were judged to be possibly relevant for final inclusion (Figure 1). Of the 87 full-text studies reviewed, 29 were included for data extraction. These included 17 studies pertaining to NRH in the native liver resulting in liver transplant or listing for liver transplant (6, 7, 14–29), and 11 studies pertaining to the development of NRH after liver transplantation (30–40).

### NRH as an indication for liver transplant

A summary of included studies is shown in Table 1. There were 113 liver transplants performed for patients with NRH. All patients had portal hypertension, and in 112, portal hypertension was the primary indication for transplant. In one patient, HCC was the primary indication for transplant (20). Of the studies that reported the incidence of NRH of the native liver amongst all liver transplant recipients, pooled incidence of NRH was 0.9% (16, 24, 33). The possible cause of NRH was highly variable, and a suggested cause of NRH was identified in 52% of cases. Five-year graft survival for studies reporting such data ranged from 73%–78% (3, 22, 24). with 5-year patient survival ranging from 73%–90% amongst NRH patients (3, 22, 24). We found reports of five patients being transplanted for NRH secondary to common variable immunodeficiency (CVID) (6, 27, 28). The outcomes for these patients were poor. All patients developed recurrent NRH after transplant, albeit at variable duration. Two developed recurrent NRH very early after transplant leading to death or retransplant; another developed recurrence of NRH much later at 5 years and required a retransplant; and two others developed late NRH, which was asymptomatic. There were eight cases of reported recurrence of NRH (6, 21, 22, 24, 27, 29). Unfortunately, there were variable details on the severity or timing of recurrent NRH, with recurrence developing anywhere from 3 months to 14 years post-transplant and in severity ranging from asymptomatic (discovered on protocol biopsy) to requiring re-transplantation.

**Table 1:** NRH as indication for liver transplant.

Author	Year	Study Interval	Study Design	Population	Possible Cause of NRH	Complications of NRH	Pre-transplant treatment	Graft survival	Patient survival	NRH recurrence
McDonald et al.	1990	1986	Case report	1 patient transplanted for NRH	PVT	Ascites, encephalopathy	–	Died 4 months post-transplant from infection		–
Elariny et al.	1994	1990	Case report	1 patient transplanted for NRH	Immunosuppression for previous kidney transplant	Varices, ascites	Variceal sclerotherapy	Good graft function and alive at 2 years		–
Radomski et al.	2000	1982-1997	Case series	4 (of 355 total transplants)	–	Varices (3), ascites (1), ecephalopathy (1)	TIPS (1)	100% at 2–4 year follow up		–
Devarbhavi et al.	2001	1983–2000	Case series	6 patients transplanted for NRH	Rheumatoid arthritis (4)	Ascites, GI bleed, encephalopathy	–	–	–	0% at up to 8 year follow up
Dumortier et al.	2001	1987–1994	Case series	8 Patients transplanted for NRH	–	Varices (8), PVT (2), thrombocytopenia (1)	Variceal sclerotherapy or banding (3), TIPS (2), surgical portocaval shunt (1)	100% at 1–8 year follow up		–
Buchel et al.	2005	–	Case report	1 patient transplanted for NRH	PVT, hyperhomocysteinemia	Varices, encephalopathy, thrombocytopenia	No	Good graft function and alive at 2 years		
Mallet et al.	2007	2003–2006	Prospective cohort	8 of 97 HIV + patients on HAART with NRH	HAART (8)	Varices (6), ascites (3), thrombocytopenia (8)	TIPS (1)	No patients transplanted, but 3 were listed for liver transplant		–
Kumari et al.	2010	–	Case report	1 patient transplanted for HCC in setting of NRH	–	HCC, ascites	TACE	Good graft function and alive at 6 months		–
Sultanik et al.	2013		Case series	4 patients transplanted for NRH	HAART (4)	Varices (4), ascites (1)	–	50% (retransplant for hepatic artery rupture and death from renoportal anastomosis thrombus)		1 case of asymptomatic NRH recurrence at 1 year
Sood et al.	2014	2007	Case report	1 patient with HCC in setting of NRH	PVT	Varices	Variceal banding, RFA	Patient listed for transplant		–
Patcha et al.	2014	2013	Retrospective cohort	13 patients transplanted for NRH	Drug (6), PVT (6), hypercoagulable disorder (2)	Ascites (10), varices (9), encephalopathy (8)	–	96% at 5 years	76% at 5 years	2/13 (1 required retransplant, 1 died)
Abraham et al.	2016	1995–2003	Retrospective cohort	11 (of 306 transplants)	PSC, PBC, autoimmune cholangitis	Splenomegaly (10), varices (9), ascites (7)	TIPS (2)	–	–	–
Meijer et al.	2017	1995–2016	Retrospective cohort	11 (of 1886 transplants)	AZA (5)	Varices (11), ascites (7), encephalopathy (4), HPS (4)	–	100%	75% (ARDS, ICH, sepsis)	1 NRH recurrence at 14 years
Johnson et al.	2021		Case report	1 patient transplanted for NRH	Autoimmune lympoproliferative disorder	Varices, HPS	TIPS	Good graft function and alive at 25 days		–
Hercun et al.	2021		Case series	3 patients transplanted for NRH	CVID (3)	Ascites, varices, HPS		3/3 patients had recurrent NRH. 1 died from graft and multisystem organ failure; 1 was retransplanted at 5 years; 1 was asymptomatic with from NRH		
Penrice et al.	2022	2002–2017	Retrospective cohort	167 patients with NRH (22 transplanted)	Hematologic (19%), rheumatologic (11%), drug related (10%), unknown (56%)	Splenomegaly (38%), ascites (31%), varices (23%)	TIPS (5%)	13% of patients with NRH were transplanted. No follow up after transplant.		
O’Neil et al.	2021		Case report	1 patient transplanted for NRH	CVID	Ascites, varices, encephalopathy	–	Transplanted for NRH, then re-transplanted for HAT at 10 days. 3 months after re-transplant, patient developed recurrent NRH.		
Bonatti et al.	2023		Case report	1 patient transplanted for NRH	CVID	–	–	Transplanted for NRH, developed recurrent NRH at 18 months. Has persistent NRH, but functioning graft at 10 years.		

PVT, portal vein thrombus; TIPS, transjugular intrahepatic portosystemic shunt; HIV, human immunodeficiency virus; HAART, highly active antiretroviral therapy; HCC, hepatocellular carcinoma; TACE, transarterial chemoembolization; RFA, radiofrequency ablation; PSC, primary sclerosing cholangitis; PBC, primary biliary cirrhoris; AZA, azathioprine; HPS, hepatopulmonary syndrome; ARDS, acute respiratory distress syndrome; ICH, intracranial hemorrhage; CVID, common variable immune deficiency.

### NRH development after liver transplant

The mean incidence of NRH among liver transplant recipients was 2.9% (Tables 2, 3) (32, 34, 36, 38–40). Timing of NRH development was highly variable, and NRH was often discovered on protocol surveillance biopsies post-transplant in asymptomatic patients (33, 36, 40). For patients with NRH, those who were asymptomatic ranged anywhere from 34%–91%. Symptoms were largely those typically associated with portal hypertension. There was also significant heterogeneity among studies in rates of exposure to AZA and vascular abnormalities (known risk factors for NRH).

**Table 2:** NRH development after liver transplant.

Author	Year	Study Interval	Study Design	Population	Protocol Bx	AZA	Vascular abnormalities	Asymptomatic	NRH timing
Gane	1994	1981–1992	Retrospective cohort	9 liver transplant recipients who developed NRH	–	100%	11% PVT	–	6–144 months
Sebagh et al.	1995	1988–1989	Case series	2 liver transplant recipients who developed NRH	No	100%	–	–	2–3 years
Breen et al.	2005	2003	Prospective cohort	65 liver transplant recipients (2 got NRH)	–	100%	–	–	–
Devarbhavi et al.	2007	1991–2004	Retrospective cohort	14 (of 1191 total liver transplants)	Yes	36%	Hepatic vein narrowing (36%), increased hepatic artery resistance (21%), portal vein thrombus (14%)	50%	50% <4 years
Malik et al.	2007	1990–2005	Retrospective cohort	76 liver transplant patients who developed NRH (of 3717)	–	–	Portal vein or hepatic artery problem (6.5%)	90%	2–656 weeks
Buster et al.	2007		Case report	1 liver transplant patient who developed NRH	No	100%	PVT	–	14 years
Oo et al.	2010	2000	Case—control	151 liver transplant recipients who underwent biopsy (33% had NRH)	Yes	98%	PVT (1)	–	3–164 months
Alhosh et al.	2014		Case report	1 liver transplant recipient who developed NRH	No	0%	–	–	2 years
Gonzalez et al.	2018	2000–2018	Retrospective cohort	17 pediatric liver transplant recipients who got NRH (of 206 total transplants)	No	0%	47%	–	47% <4 years
Chen et al.	2022	1988–2018	Retrospective cohort	49 liver transplant recipients who developed NRH (of 3711 total transplants)	No	14%	6% artery, 24% portal vein, 4% cava abnormality	34.6%	49% <4 years
Kounis	2023	2004–2018	Case—control	85 liver transplant recipients who developed NRH (of 1648 total transplants)	Yes	12%	PVT (7%)	91%	8.4 years median time to NRH symptoms

AZA, azathioprine; Bx, biopsy; NRH, nodular regenerative hyperplasia; PVT, portal vein thrombus.

**Table 3:** NRH after liver transplant outcomes.

Author	Vascular intervention	Complications of NRH	Graft failure	Cause of graft failure	Patient survival	Cause of death
Gane et al	–	All had elevated ALP; withdrawal of AZA improved LFTs in 4	44%	Progressive liver dysfunction	–	–
Sebagh et al.	–	Ascites (50%)	100%	Biliary strictures (1), NRH (1)	–	–
Devarbhavi et al.	Hepatic vein angioplasty (1), splenorenal shunt (1); NRH on biopsy resolved in both after intervention	Ascites (50%), variceal bleeding (29%)	29%	HAT (1), NRH/PVT (3)	71%	Death during retransplant (1), HAT after retransplant (1), mesenteric thrombosis (1), unrelated to transplant (1)
Malik et al.		Ascites or variceal bleeding (10%)	6.5%	Recurrent disease (2), graft failure (3)	78% after 8 years	Death from graft failure (1), unrelated to graft (16)
Buster et al	–	Variceal bleeding	–	–	–	–
Oo et al.	–	–	–	–	82% (non-NRH) vs. 72% (NRH)	–
Alhosh et al.	–	Hepatopulmonary syndrome	100%	NRH/HPS	100%	–
Gonzalez et al.	Portal vein angioplasty (1), hepatic vein angioplasty (1)	Portal hypertension symptoms (29%)	24%	Hepatic vein stenosis (1), portal HTN (1), chronic rejection (1), autoimmune hepatitis (1)	88%	CMV pneumonitis (1), Pancolitis secondary to portal HTN (1)
Chen et al.	16.3%	65% had complications of portal HTN	22%	–	63%	–
Kounis et al.	6% surgical portocaval shunt or TIPS	Ascites (16%), varices (11%), encephalopathy (3%)	5-year graft survival 94% control vs. 92% NRH	56% of retransplants thought to be secondary to NRH	5-year patient survival 93% vs. 84% control	3.5% of patients died from NRH complication

NRH, nodular regenerative hyperplasia; ALP, alkaline phosphatase; AZA, azathioprine; LFTs, liver function tests; HAT, hepatic artery thrombus; PVT, portal vein thrombus; HTN, hypertension; TIPS, transjugular intrahepatic portosystemic shunt.

Complications of NRH development after liver transplantation ranged in severity from changes in liver function to the development of portal hypertension. There are reports of intervening on vascular abnormalities, with variable follow-up as to the persistence or resolution of NRH (33, 38, 39). Some patients required endovascular or surgical portocaval shunts for the treatment of portal hypertension (40). Of studies with larger cohorts of patients, graft failure ranged anywhere from 6%–44% and patient survival ranged from 63%–93% (30, 33, 34, 36, 39, 40).

## Discussion

Autopsy studies suggest NRH is a relatively common phenomenon (incidence of up to 2%) that remains indolent in the majority of patients; however, NRH is a rare indication for liver transplantation to treat the symptoms of associated portal hypertension. Given the rarity of NRH, in this study, we systematically reviewed the literature on NRH as it relates to liver transplantation. Although NRH is rare as an indication for liver transplantation, studies that followed patients long-term demonstrated that graft and patient survival were acceptable and comparable to outcomes for liver transplantation for other indications. Most graft losses and death were for reasons unrelated to NRH, and recurrence was uncommon.

All reports of NRH were diagnosed by histology. However, many did not report which histologic criteria were required for NRH diagnosis. Similar to past reports, there was a diverse array of hypothesized causes of NRH that were an indication for patients to undergo liver transplantation (3). As has been demonstrated previously, portal vein thrombus (PVT) was sometimes found in NRH patients though studies did not report on the temporal relationship of PVT and NRH, making it challenging to draw conclusions about the causative relationship of PVT to NRH. We did not identify any trends between patients with and without an identifiable cause of NRH. One specific cause, however, stood out. The outcomes after transplant for CVID were exceptionally poor, as outlined above with a 100% NRH recurrence rate and 3/5 having either a recipient death or graft failure. Although this is a limited sample size, this scenario should be approached with caution given the high NRH recurrence rates in this specific patient population.

Another unique scenario identified involves the development of hepatocellular carcinoma (HCC) in the setting of NRH, as NRH is not traditionally thought to be a premalignant lesion. We found one report of a liver transplant performed for HCC, and the explanted liver showed NRH, without cirrhosis (20). This HCC was initially treated with transarterial chemoembolization (TACE), and the patient had a satisfactory outcome. In an autopsy series including five patients with NRH and HCC, all patients had TACE therapy (41). It is unclear in any of these cases if HCC developed in a background of NRH or if TACE treatment of HCC in a non-cirrhotic liver led to NRH development. This is plausible given the known risk of vascular abnormalities causing NRH. However, there has been a report of a patient being followed up for NRH having an HCC discovered on a surveillance ultrasound (29).

It is not well described in the literature when to transplant patients with NRH. Some studies report attempts of managing portal hypertension and its stigmata with usual best-practice medical modalities—endoscopic treatment of varices, ascites management with diuretics, and pharmacologic management of encephalopathy. Meanwhile, others report the use of transjugular intrahepatic portosystemic shunt (TIPS) for managing NRH-associated portal hypertension. It is interesting that therapies such as TIPS were not used more aggressively pre-transplant in this patient population. However, our search may not have encompassed all uses of TIPS in NRH, as we focused on liver transplantation and NRH. For example, successful long-term treatment of patients with portal hypertension from NRH is described for ascites and variceal bleeding management, and these patients may successfully avoid liver transplantation (42).

After a liver transplant, NRH occurrence is more common than we expect. It is difficult to predict who will get NRH after transplant, and there is no consensus about the causes of NRH after transplantation. One study identifies older donors as a risk factor for NRH (40), while other studies are not able to identify any independent risk factors. The timing of when NRH develops is also unpredictable and the prognosis of early vs. late NRH development is not well known. There have been conflicting reports, with some studies suggesting early NRH is more likely to lead to a negative outcome of graft loss or patient death, and others have found the opposite to be true, with late NRH (>4 years) being a predictor of a poor outcome (33, 39). Additionally, it is difficult to know what to do with the information if a recipient has NRH discovered on biopsy. Much of the larger previous series were done at centers that were standardly performing protocol biopsies at various intervals after transplant. The incidence of clinically meaningful NRH is likely much lower than what was reported in these studies. This series of protocol biopsies discovering asymptomatic NRH suggests that likely many cases of NRH never go on to develop portal hypertension or have a meaningful impact on graft function.

Many studies included patients with exposure to AZA as a part of their immunosuppression. This is much less relevant for modern immunosuppression regimens; in fact, it has been documented that withdrawal of AZA can reverse some cases of NRH in the transplanted liver (30). However, in a population of pediatric liver transplant recipients with no AZA exposure, an NRH incidence of 8% was observed, suggesting NRH can develop in immunosuppressed patients in the absence of AZA. However, this was a pediatric population with a high rate of vascular abnormalities being discovered post-transplant which could explain why NRH was common in this series (38). Another important point is that NRH was discovered on protocol biopsy of the transplanted liver in many of these studies. This calls into question whether the incidence of clinically relevant NRH is overestimated in these series. This point is further highlighted by the wide range of severity of NRH from asymptomatic to severe portal hypertension requiring liver re-transplantation.

It is challenging to know what the best management strategy is when NRH is discovered. If there is an identified medical or vascular cause thought to be contributing to the NRH, this should obviously be immediately discontinued or reversed (e.g., stopping any offending medications or evaluating for any vasculature anomalies that can be intervened on). For more severe cases, we cannot draw any conclusions from the available literature regarding standard management. However, there have been cases of successfully managing portal hypertension with TIPS, and patients have successfully been retransplanted. Not surprisingly, patients with NRH and symptomatic portal hypertension have a higher mortality rate than those without portal hypertension (39, 40).

Our review has several limitations. There is significant heterogeneity of patient populations and endpoints measured. This heterogeneity makes it difficult to directly compare different studies. There are also a wide range of time periods included. Older studies may be less relevant as practice patterns have changed, with less reliance on AZA-based immunosuppression and protocol biopsies after liver transplant. Specifically for the patient population that developed NRH in a transplanted liver, the larger series have very limited donor data which may be an important variable in NRH development. A challenge in interpreting the current data is that little is known about the natural history of NRH. It seems, especially based on incidentally discovered, asymptomatic NRH that at least some cases of NRH do not cause significant problems. Still, other cases cause severe portal hypertension. How to best navigate this spectrum of disease severity without knowing the natural history will continue to be a challenge for clinicians. Finally, as described above, the rarity of the pathology, variable severity, and treatments make it difficult to draw conclusions regarding optimal treatment plans.

## Data Availability

The original contributions presented in the study are included in the article/**Supplementary Material**, further inquiries can be directed to the corresponding authors.
