# Supplementary material for: Nodular regenerative hyperplasia and liver transplantation: a systematic review
Source: Front Transplant. 2023 Sep 6;2:1221765. doi: 10.3389/frtra.2023.1221765 (PMC11235372; doi:10.3389/frtra.2023.1221765)
Supplement: Supplementary file 1 [file Datasheet1.docx]

| "Kidney Transplantation"[Mesh] OR "Organ Transplantation"[Mesh] OR "Liver Transplantation"[Mesh] OR "Pancreas Transplantation"[Mesh] OR (transplant*[tiab] AND (kidney*[tiab] OR liver[tiab] OR "solid organ"[tiab] OR renal[tiab] OR pancreas[tiab] OR intestin*[tiab])) | Focal Nodular Hyperplasia"[Mesh]) OR "Nodular regenerative Hyperplasia"[tiab] |  |
| --- | --- | --- |

**PubMed**

**(((("Kidney Transplantation"[Mesh]) OR "Organ Transplantation"[Mesh]) OR "Liver Transplantation"[Mesh]) OR "Pancreas Transplantation"[Mesh] OR (transplant*[tiab] AND (kidney*[tiab] OR liver[tiab] OR "solid organ"[tiab] OR renal[tiab] OR pancreas[tiab] OR intestin*[tiab] OR hepat*[tiab] OR organ[tiab]))) AND (("Focal Nodular Hyperplasia"[Mesh] OR "Nodular regenerat* Hyperplasia"[tiab] OR "regenerative hyperplasia"[tiab] OR "recurrent hyperplasia"[tiab]))**

165 results no limits applied

**CINAHL**

( (MH "Kidney Transplantation") OR (MH "Pancreas-Kidney Transplantation") OR (MH "Liver Transplantation") OR (MH "Organ Transplantation") OR (MH "Pancreas Transplantation") ) OR TI ( (transplant* AND (kidney* OR liver OR "solid organ" OR renal OR pancreas OR intestin*) ) OR AB ( (transplant* AND (kidney* OR liver OR "solid organ" OR renal OR pancreas OR intestin*) )

AND

TI ( "Focal Nodular Hyperplasia" OR "Nodular regenerative Hyperplasia" ) OR AB ( "Focal Nodular Hyperplasia" OR "Nodular regenerative Hyperplasia" )

16 results no limits applied

**Scopus**

( TITLE-ABS-KEY ( {Focal Nodular Hyperplasia} OR "Nodular regenerat* Hyperplasia" OR "regenerative hyperplasia" ) AND TITLE-ABS-KEY ( transplant* AND ( kidney* OR liver OR "solid organ" OR renal OR pancreas OR intestin* ) ) )

351 results no limits applied

**Web of Science**

"Focal Nodular Hyperplasia" OR "Nodular regenerat* Hyperplasia" OR "regenerative hyperplasia" (Topic) and transplant* AND ( kidney* OR liver OR "solid organ" OR renal OR pancreas OR intestin* ) (Topic)

436 results no limits applied

**Cochrane**

## 1 matching "Focal Nodular Hyperplasia" OR "Nodular regenerat* Hyperplasia" OR "regenerat* hyperplasia" in Title Abstract Keyword AND transplan* in Title Abstract Keyword - (Word variations have been searched)

Bottom of Form
